# Assessment of bone involvement by DXA and HR-pQCT in patients with X-linked hypophosphatemia

**DOI:** 10.1007/s00774-026-01748-6

**Published:** 2026-08-11

**Authors:** Iara Marcelino Sant′Ana, Maria Caroline Alves Coelho, Joyce Cantoni, Leandro Kasuki, Maria Lucia Fleiuss Farias, Miguel Madeira

**Affiliations:** 1 Instituto Estadual de Diabetes e Endocrinologia Luiz Capriglione (IEDE), Rio de Janeiro, RJ Brazil; 2 Division Endocrinology, Universidade Federal do Rio de Janeiro (UFRJ), Rua Professor Rodolpho Paulo Rocco, 255, Ilha do Fundão, Rio de Janeiro, RJ 21941-617 Brazil; 3 Universidade do Estado do Rio de Janeiro (UERJ), Rio de Janeiro, RJ Brazil

**Keywords:** Hypophosphatemic rickets, X-linked hypophosphatemic rickets, High-resolution peripheral quantitative computed tomography (HR-pQCT), Dual energy X-ray absorptiometry (DXA)

## Abstract

**Introduction:**

X-linked hypophosphatemic rickets (XLH) is a rare disease and its natural history affects the quality of life of patients, as it leads to bone deformities and architectural and qualitative changes in the bone.

**Materials and methods:**

Sixteen XLH patients were selected from referral hospitals in Rio de Janeiro (12 women and four men) and compared to 16 healthy age- and sex-matched control individuals. All XLH were receiving calcitriol and oral phosphate supplements. Bone assessment was performed by high-resolution peripheral quantitative computed tomography (HR-pQCT) and dual-energy X-ray absorptiometry (DXA).

**Results:**

XLH patients showed higher lumbar spine BMD Z-score, but lower 33% distal radius BMD and Z-score values as compared to control group. Low BMD for age (Z-score ≤ -2.0 SD) was only detected in the XLH group: specifically in 3 of the 5 postmenopausal women, 3 of the 7 premenopausal women, and 2 of the 4 men. HR-pQCT evaluation showed lower total vBMD at both peripheral sites and impaired trabecular parameters, particularly at the distal tibia, after correction for multiple comparisons.

**Conclusion:**

Our study sheds light on the involvement of both trabecular and cortical compartments in the XLH group, despite conventional treatment. We also emphasize the limitation of DXA in assessing central sites in the XLH group. The 33% radius may represent a useful complementary skeletal site for DXA assessment, particularly when HR-pQCT is not available.

## Introduction

The most common genetic form of rickets and osteomalacia is X-linked hypophosphatemic rickets (XLH), caused by a loss-of-function mutation in the phosphate-regulating gene with homologies to endopeptidases on the X chromosome *(PHEX)*. This mutation leads to increased serum fibroblast growth factor 23 (FGF23) levels, thus causing hyperphosphaturia and hypophosphatemia [1]. XLH is a rare disease with an estimated incidence between 1:20,000 and 1:60,000 live births [2, 3].

The diagnosis of XLH relies on the combination of clinical, radiographic, biochemical and genetic features, whenever possible. In other words, this involves signs of rickets and/or osteomalacia in association with hypophosphatemia and renal phosphate wasting in the absence of vitamin D deficiency [4].

Most clinical screenings in children are performed because of a known family history of XLH. Suspicion lies with a progressive bowing of the lower extremity when the child begins to walk, reduced growth velocity, associated with bone and/or joint pain. Manifestations in adults consist of persistent bone pain, early osteoarthritis, pseudofractures, enthesopathy, and/or periodontitis [5].

Treatment in adults should be considered in symptomatic patients, patients undergoing orthopedic or dental surgery and women during pregnancy and lactation. Treatment with oral phosphate salts and active vitamin D analogs has incomplete efficacy and potential risks because it does not directly target the underlying pathophysiology of the disease. Burosumab, a recombinant human monoclonal antibody against FGF23, has proven its efficacy in phase 2 and phase 3 clinical trials in adult patients with XLH, but currently its recommendation as first line or second line treatment differ among the countries [6].

Dual-energy X-ray absorptiometry (DXA) is the most common technique used to evaluate areal bone mineral density (aBMD). Evidence from studies assessing aBMD by DXA in XLH has shown a trend toward higher BMD in the axial skeleton and lower BMD in the appendicular skeleton [7–10]. However, the large variability between anatomical and anthropometric parameters in XLH patients interferes with DXA results, which therefore may not reflect the real bone status. However, high-resolution peripheral quantitative computed tomography (HR-pQCT) is only slightly affected by these factors and has been used to characterize the effects of XLH on the volumetric bone mineral density (vBMD) and bone microarchitecture, particularly in XLH adults [11].

Due to the paucity of published data, the aim of this study was to use DXA and HR-pQCT to assess bone parameters in adult patients with XLH compared with healthy controls.

## Materials and methods

### Subjects

The study protocol was approved by the Ethics Committee of Instituto Estadual de Diabetes e Endocrinologia Luiz Capriglione (IEDE), and informed consent was obtained from all patients and controls.

Patients were selected from two referral hospitals in Rio de Janeiro: Endocrinology Service of the Hospital Universitário Clementino Fraga Filho (HUCFF-UFRJ) and IEDE. The inclusion criteria required a confirmed diagnosis of XLH by genetic testing, although patients with genetic variant of uncertain significance or untested relatives of patients with identified mutations were also included, provided that their clinical and laboratory findings were compatible with the diagnosis. All participants, XLH patients and control subjects, were 18 years of age or older. The exclusion criteria were inability to cooperate with the examinations, other coexisting osteometabolic diseases—not related to hypophosphatemic rickets or its treatment; use of medications or diseases that may interfere with bone health (bisphosphonates, calcitonin, raloxifene, denosumab, teriparatide, romosozumab, hypercortisolism, hyperthyroidism); pregnant women. XLH group comprised four men and twelve women (five postmenopause and seven premenopause). All patients were undergoing conventional treatment (oral phosphate salts and active vitamin D analogs) at the time of the study. They were compared with healthy control subjects matched for age, sex, and, in the case of women, menopausal status. The control group was composed of healthy employees and volunteers of the HUCFF-UFRJ. The exclusion criteria for the control group were the presence of osteometabolic diseases, previous fractures, chronic diseases, smoking, alcoholism, and any use of medications that interfere with bone metabolism.

### DXA

The areal bone mineral density (BMD) at the lumbar spine, the hip, and the 33% radius were analyzed by DXA (GE Lunar Prodigy Advance, GE Healthcare, Madison, WI, USA). Results are expressed as absolute values and standard deviation in relation to young adults (T-score) and standard deviation in relation to people in the same gender and age group (Z-score). The use of the T-score is recommended only for postmenopausal women and for men aged 50 or over. In premenopausal women and men under 50, the use of the Z-score is recommended, with the following classification:

- “below the estimated value for the age group”: Z-score ≤ − 2.0 SD;
- “within the estimated value for the age group”: Z-score > − 2.0 SD.

A T-score between − 1.0 and − 2.5 SD indicates osteopenia, while a T-score of − 2.5 SD or lower indicates osteoporosis [12, 13].

Lumbar spine DXA scans were reviewed for the presence of degenerative changes, including osteophytes and ligamentous ossification. Vertebrae affected by significant artifacts were excluded from analysis according to ISCD recommendations whenever possible.

### HR-pQCT

Volumetric BMD and bone microstructure were evaluated by HR-pQCT using the Xtreme CT scanner (SCANCO Medical AG, Brüttisellen, Switzerland) at the non-dominant distal radius and distal tibia. The following parameters were calculated: vBMD for the entire region (total vBMD, mg hydroxyapatite/cm^3^), trabecular region (Tb.vBMD, mg hydroxyapatite/cm^3^), and cortical region (Ct.vBMD, mg hydroxyapatite/cm^3^); trabecular bone volume to tissue volume fraction (Tb.BV/TV); trabecular number (Tb.N, mm^−1^); trabecular thickness (Tb.Th, mm); trabecular separation (Tb.Sp, mm); trabecular network inhomogeneity (SD.1/Tb.N, mm); and cortical thickness (Ct.Th, mm).

### Laboratory tests

Blood tests, performed in the same period of imaging exams, included total and bone Alkaline Phosphatase (ALP and BALP) as markers of disease activity, Parathyroid Hormone (PTH) to assess hyperparathyroidism, calcium, phosphorus, albumin, creatinine, calcitriol and FGF23.

### Statistical analyses

The results are presented as frequencies and percentages for categorical variables and as mean ± standard deviation or median (interquartile range) for numerical variables depending on the sample distribution. Fisher's exact test was used to analyze categorical data. The Mann–Whitney U test or Student's t test was used to analyze numerical variables, as appropriate. Effect sizes were calculated for between-group comparisons. For non-parametric comparisons, Cliff’s delta (δ) was used, whereas effect size r was reported when appropriate. The analyses were carried out with SPSS 20.0 program for MAC (IBM, Chicago, IL, USA).

To account for multiple comparisons, false discovery rate (FDR) correction was performed using the Benjamini–Hochberg procedure. Given the exploratory nature of the subgroup analyses and the limited sample size, FDR-adjusted *p*-values are presented in the tables and interpreted alongside effect sizes. The level of significance was set at FDR-adjusted *p* < 0.05.

## Results

Our sample consisted of 16 XLH patients and 16 healthy controls matched for age, sex, and menopausal status. Although mean BMI was numerically higher in the XLH group (29.87 ± 9.87 vs 26.23 ± 5.40 kg/m^2^), this variable was not specifically matched, and mean height was significantly lower in XLH patients (146.75 ± 8.10 vs 163.31 ± 6.56 cm, *p* < 0.0001).XLH and control group included four men, five women in menopause and seven women in pre-menopause. The average age at diagnosis of XLH was 14.75 years ± 16.72, and all patients received conventional treatment with phosphate salts and calcitriol. Ten patients started treatment in childhood (3.4 years ± 2.17), one patient in adolescence (13 years), and five patients in adulthood (37.8 years ± 7.12). Four XLH patients had a history of previous orthopedic surgery (one male, one menopausal and two premenopausal patients).

Biochemical evaluation of XLH patients is shown in Table 1. Serum PTH was elevated in three patients (two men and one postmenopausal woman), and total alkaline phosphatase and bone alkaline phosphatase were elevated in five patients (two men, two premenopausal women, and one menopausal woman).

**Table 1:** Biochemical characteristics of the whole XLH group

	Mean	SD	Median	Interquartile range	Reference values
Calcium (mg/dL)	9.14	0.56		8.7–9.4	8.6–10.5
Phosphorus (mg/dL)	2.35	0.29	2.3	2.15–2.5	2.5–4.5
Creatinine (mg/dL)	0.64	0.21	0.61	0.46–0.79	0.72–1.25
PTH (pg/mL)	57.3	28.5	58.9	33.9–65.6	18.5–88
Calcitriol (pg/mL)	51.8	18.2	45.8	37.6–67.1	19.9–79.3
FGF23 (KRU/L)	252.7	325.3	103.5	71.8–251.5	26–110
Alkaline phosphatase (U/L)	109.4	33.6	101	90–124	36–110
BALP (mcg/L)	24.5	15.7	18.3	15.1–26.4	Men: 5.5–22.9 Pre menopause: 4.9–26.6 Post menopause: 5.2–24.4

*PTH* parathyroid hormone; *FGF23* phosphaturic growth factor 23; *BALP* bone alkaline phosphatase

DXA evaluation showed differences between XLH and control subjects, as seen in Tables 2 and 3. After FDR correction, XLH patients had lower 33% radius BMD and Z-score values, whereas lumbar spine Z-score showed a borderline FDR-adjusted value and no difference was observed at the hip. Half of the XLH patients were classified as having “low BMD for age” based on measurements at the 33% radius, and one postmenopausal woman with XLH was classified as having osteoporosis.

**Table 2:** Comparison of DXA parameters between XLH and control groups

	XLH (*N *= 16)	Control (*N *= 16)	FDR-adjusted *p*-value
Lumbar spine BMD (g/cm^2^)	1.368 (1.274–1.478)	1.258 (1.094–1.423)	0.101
Lumbar spine Z-score	1.6 (0.8–2.5)	− 0.15 (− 0.58–1.35)	0.050
Total hip BMD (g/cm^2^)	1.036 (0.848–1.115)	1.115 (0.963–1.175)	0.274
Total hip Z-score	0.20 (− 0.8–1)	0.25 (− 0.30–0.75)	0.427
33% radius BMD (g/cm^2^)	0.737 (0.673–0.794)	0.81 (0.770–0.870)	0.021
33% radius Z-score	− 2.0 (− 2.3 to − 1.6)	− 0.55 (− 1.28 to − 0.15)	<0.006
Physical activity	4/16 = 25%	6/16 = 37.5%	
Smoking	0	0	
Diabetes	0	0	

Data are expressed as median (interquartile range). Displayed *p*-values were adjusted using the Benjamini–Hochberg false discovery rate (FDR) procedure

**Table 3:** DXA analysis of XLH and control subgroups: men, postmenopausal and premenopausal women

DXA parameters	Postmenopause XLH (*n *= 5)	Postmenopause control (*n *= 5)	Premenopause XLH (*n *= 7)	Premenopause control (*n *= 7)	Men XLH (*n *= 4)	Men control (*n*=4)
age	58 (55–63)	58 (55–63)	23 (21–44)	23 (21–44)	28 (25–39)	28 (25–39)
BMI	30.0 (26.8–33.0)	26.3 (23.8–28.8)	28.5 (25.0–31.0)	24.8 (22.0–27.2)	31.5 (28.5–35.0)	27.8 (25.2–30.5)
LS BMD (g/cm^2^)	1.223 (1.108–1.688)	1.027 (0.937–1.111)	1.368 (1.359–1.439)	1.258 (1.120–1.375)	1.460 (1.354–1.503)	1.434 (1.422–1.441)
LS Z-score	2.0 (0.23–5.2)	− 0.7 (− 1.58 to − 0.43)	1.4 (0.9–2.4)	− 0.15 (− 0.63–1.18)	1.4 (1.35–2.73)	1.45 (1.35–1.58)
LS T-score	0.25 (− 0.65–4.23)	− 1.3 (− 2.1 to − 0.65)	NA	NA	NA	NA
TH BMD (g/cm^2^)	0.916 (0.676–0.965)	0.935 (0.825–1.130)	1.074 (0.848–1.092)	1.115 (0.949–1.181)	1.197 (1.036–1.213)	1.19 (1.12–1.28)
TH Z-score	− 0.8 (− 1.0–0.3)	− 0.25 (− 1.03–1.13)	0.3 (− 1.15–1.1)	0.25 (− 0.3–0.8)	0.2 (− 0.2–1.0)	0.70 (0.35–1.2)
TH T-score	− 0.7 (− 2.6 to − 0.3)	− 0.6 (− 1.48–0.25)	NA	NA	NA	NA
33% R BMD (g/cm^2^)	0.707 (0.439–0.774)	0.814 (0.712–0.834)	0.713 (0.671–0.737)	0.810 (0.762–0.897)	0.824 (0.796–0.865)	0.87 (0.77–0.99)
33% R Z-score	− 2.05 (− 3.83 to − 0.95)	− 0.75 (− 0.9 to − 0.08)	− 1.9 (− 2.4 to − 1.6)	− 1.4 (− 1.6 to − 0.08)	− 1.8 (− 2.1 to − 1.38)	−0.35 (− 0.43 to − 0.18)
33% R T-score	− 2.0 (− 5.03 to − 1.3)	− 0.8 (− 1.98 to − 0.53)	NA	NA	NA	NA
Low BMD for age (N)	3	0	3	0	2	0
Osteoporosis (N)	1	0	NA	NA	NA	NA

Data expressed as median (interquartile range). BMD Z-scores were evaluated in all individuals, while T-scores were only considered in postmenopausal women

*NA* not applicable; *LS* lumbar spine; *TH* total hip; 33% *R* radius; 33%. Low BMD for age (Z-score ≤− 2.0 SD). Osteoporosis (lowest T-score ≤− 2.5 SD)

HR-pQCT evaluation at the distal radius and distal tibia identified lower total vBMD at both peripheral sites in XLH patients compared with controls (Table 4). After FDR correction, the most consistent differences were observed in the trabecular compartment of the distal tibia, including lower Tb.vBMD, Tb.BV/TV and Tb.Th. At the radius, total vBMD remained significantly lower, while Ct.Th and Tb.Th showed borderline FDR-adjusted values.

**Table 4:** Bone parameters of HR-pQCT of the tibia and radius in XLH group and controls

	XLH group (*N *= 16)	Control group (*N *= 16)	FDR *p*-value
*Distal radius*			
Total vBMD (mg HA/cm^3^)	267.00 (223.90–319.73)	352.50 (302.30–430.03)	0.027
Ct.vBMD (mg HA/cm^3^)	875.60 (800.25–909.30)	925.90 (884.03–955.90)	0.124
Tb.vBMD (mg HA/cm^3^)	137.90 (111.70–178.65)	172.75 (121.85–194.80)	0.195
Tb.BV/TV	0.115 (0.093–0.149)	0.144** (**0.101–0.163)	0.195
Ct.Th (mm)	0.660 (0.473–0.768)	0.875 (0.685–1.048)	0.051
Tb.N (mm⁻^1^)	1.765 (1.485–2.253)	1.945 (1.555–2.040)	0.624
Tb.Th (mm)	0.060 (0.054–0.073)	0.073 (0.063–0.081)	0.052
Tb.Sp (mm)	0.499 (0.379–0.591)	0.432 (0.413–0.576)	0.507
SD.1/Tb.N (mm)	0.228 (0.152–0.371)	0.183 (0.164–0.276)	0.439
*Distal tibia*			
Total vBMD (mg HA/cm^3^)	249.10 (195.35–317.30)	338.85 (279.48–392.90)	0.040
Ct.vBMD (mg HA/cm^3^)	911.75** (**883.53–956.45)	930.60 (898.48–949.05)	0.594
Tb.vBMD (mg HA/cm^3^)	93.15 (45.78–146.70)	169.35 (129.80–205.38)	0.009
Tb.BV/TV	0.078 (0.039–0.122)	0.141 (0.109–0.171)	0.009
Ct.Th (mm)	1.180 (0.930–1.443)	1.295 (1.155–1.503)	0.348
Tb.N (mm⁻^1^)	1.240 (0.820–1.828)	1.680 (1.510–1.943)	0.056
Tb.Th (mm)	0.057 (0.046–0.073)	0.084 (0.070–0.096)	0.018
Tb.Sp (mm)	0.757 (0.487–1.171)	0.522 (0.434–0.596)	0.052
SD.1/Tb.N (mm)	0.446 (0.215–0.745)	0.245 (0.187–0.272)	0.052

Data are expressed as median (interquartile range). Displayed *p*-values were adjusted using the Benjamini–Hochberg false discovery rate (FDR) procedure

We compared patients diagnosed and treated before the age of five with those diagnosed and treated in adolescence or adulthood. All patients were receiving conventional treatment. Patients with delayed diagnosis and treatment initiation showed a pattern of poorer trabecular microarchitecture, particularly at the radius. Although these differences did not remain statistically significant after FDR correction, the early-treatment group showed numerically higher radius total vBMD, Tb.vBMD, Tb.BV/TV and Tb.Th, with large effect sizes. At the tibia, Tb.Th was also numerically higher in the early-treatment group (Table 5).

**Table 5:** HR-pQCT parameters according to age at diagnosis and treatment initiation in adults with X-linked hypophosphatemia

Parameter	Early ≤ 5 years	Delayed	FDR *p*-value	Cliff’s δ
	*N *= 10	*N *= 6		
*Tibia*				
Total vBMD, mg HA/cm^3^	280.5 (233.4–346.2)	218.3 (177.3–256.5)	0.284	0.50
Ct.vBMD, mg HA/cm^3^	916.5 (887.9–964.0)	907.8 (850.0–925.8)	0.589	0.25
Tb.vBMD, mg HA/cm^3^	102.3 (86.7–152.1)	67.4 (44.7–111.6)	0.367	0.42
Tb.BV/TV, %	8.6 (7.3–12.7)	5.7 (3.8–9.3)	0.367	0.40
Ct.Th, mm	1.29 (1.12–1.56)	1.01 (0.73–1.21)	0.284	0.50
Tb.N, mm⁻^1^	1.24 (0.98–1.74)	1.20 (0.78–1.76)	0.852	0.08
Tb.Th, mm	0.072 (0.058–0.075)	0.049 (0.045–0.054)	0.155	0.67
Tb.Sp, mm	0.757 (0.501–0.954)	0.867 (0.520–1.232)	0.799	− 0.13
SD.1/Tb.N, mm	0.446 (0.215–0.549)	0.507 (0.239–0.807)	0.745	− 0.17
*Radius*				
Total vBMD, mg HA/cm^3^	281.5 (261.1–348.3)	233.1 (178.5–264.1)	0.140	0.67
Ct.vBMD, mg HA/cm^3^	892.1 (836.6–909.3)	867.7 (804.2–875.8)	0.402	0.33
Tb.vBMD, mg HA/cm^3^	158.5 (135.8–185.9)	103.3 (65.0–131.9)	0.140	0.77
Tb.BV/TV, %	13.2 (11.3–15.5)	8.6 (5.4–11.0)	0.140	0.73
Ct.Th, mm	0.70 (0.57–0.82)	0.60 (0.43–0.65)	0.284	0.45
Tb.N, mm⁻^1^	1.87 (1.65–2.27)	1.58 (1.25–1.90)	0.284	0.47
Tb.Th, mm	0.068 (0.058–0.076)	0.050 (0.043–0.058)	0.140	0.70
Tb.Sp, mm	0.472 (0.372–0.537)	0.586 (0.471–0.768)	0.284	− 0.47
SD.1/Tb.N, mm	0.206 (0.152–0.266)	0.310 (0.197–0.381)	0.402	− 0.33

Data are presented as median (interquartile range). Displayed *p*-values were adjusted for multiple comparisons using the Benjamini–Hochberg false discovery rate (FDR) procedure. Cliff’s δ was calculated as early diagnosis/treatment versus delayed diagnosis/treatment; positive values indicate higher values in the early-treatment group. For tibial HR-pQCT, data were available for 8 patients in the early-treatment group and 6 patients in the delayed-treatment group. For radius HR-pQCT, data were available for 10 and 6 patients, respectively. Significant values refer to FDR-adjusted *p*-values < 0.05

No statistically significant differences in HR-pQCT parameters were observed between patients with uncontrolled hypophosphatemia and those with normal serum phosphate levels (Table 6).

**Table 6:** Tibial and radial bone parameters using HR-pQCT in patients with XLH: uncontrolled hypophosphatemia and normal serum phosphate

Parameter	Uncontrolled P (*n *= 10)	Normal P (*n *= 6)	FDR *p*-value	Effect size (*r*)
*Tibia*				
Total vBMD (mg HA/cm^3^)	253.0 (220.0–308.0)	197.9 (164.2–268.7)	0.819	0.23
Ct.vBMD (mg HA/cm^3^)	929.2 (900.0–973.1)	883.8 (833.3–915.5)	0.684	0.39
Tb.vBMD (mg HA/cm^3^)	94.5 (46.3–119.3)	91.8 (88.4–145.7)	0.958	0.12
Tb.BV/TV (%)	8.0 (4.0–10.0)	8.0 (7.0–12.0)	0.958	0.14
Ct.Th (mm)	1.26 (1.17–1.44)	0.98 (0.65–1.07)	0.684	0.32
Tb.N (mm⁻^1^)	1.00 (0.76–1.70)	1.56 (1.42–1.85)	0.720	0.29
Tb.Th (mm)	0.060 (0.050–0.070)	0.050 (0.050–0.060)	0.958	0.08
Tb.Sp (mm)	0.94 (0.51–1.26)	0.59 (0.46–0.65)	0.766	0.26
SD.1/Tb.N (mm)	0.55 (0.22–0.84)	0.30 (0.21–0.41)	0.684	0.31
*Radius*				
Total vBMD (mg HA/cm^3^)	267.0 (258.2–316.3)	257.6 (185.3–286.1)	0.958	0.15
Ct.vBMD (mg HA/cm^3^)	892.1 (863.9–919.1)	827.7 (786.9–873.1)	0.684	0.34
Tb.vBMD (mg HA/cm^3^)	133.1 (130.0–173.7)	154.7 (96.0–160.3)	0.958	0.02
Tb.BV/TV (%)	11.0 (11.0–14.0)	13.0 (8.0–13.0)	0.958	0.03
Ct.Th (mm)	0.68 (0.63–0.82)	0.56 (0.42–0.67)	0.684	0.36
Tb.N (mm⁻^1^)	1.69 (1.58–1.95)	2.00 (1.53–2.25)	0.958	0.06
Tb.Th (mm)	0.060 (0.060–0.070)	0.060 (0.050–0.070)	0.958	0.19
Tb.Sp (mm)	0.53 (0.46–0.55)	0.44 (0.38–0.61)	0.958	0.05
SD.1/Tb.N (mm)	0.25 (0.19–0.33)	0.18 (0.15–0.34)	0.958	0.11

Data are presented as median (interquartile range). Displayed *p*-values were adjusted using the Benjamini–Hochberg false discovery rate (FDR) procedure. Effect size is expressed as r

At the tibia, patients with uncontrolled hypophosphatemia demonstrated numerically higher total and cortical volumetric bone mineral density, as well as greater cortical thickness, with small-to-moderate effect sizes. In contrast, trabecular parameters tended to be less favorable in the uncontrolled group, including lower trabecular number and greater trabecular separation and heterogeneity.

**Fig. 1:**
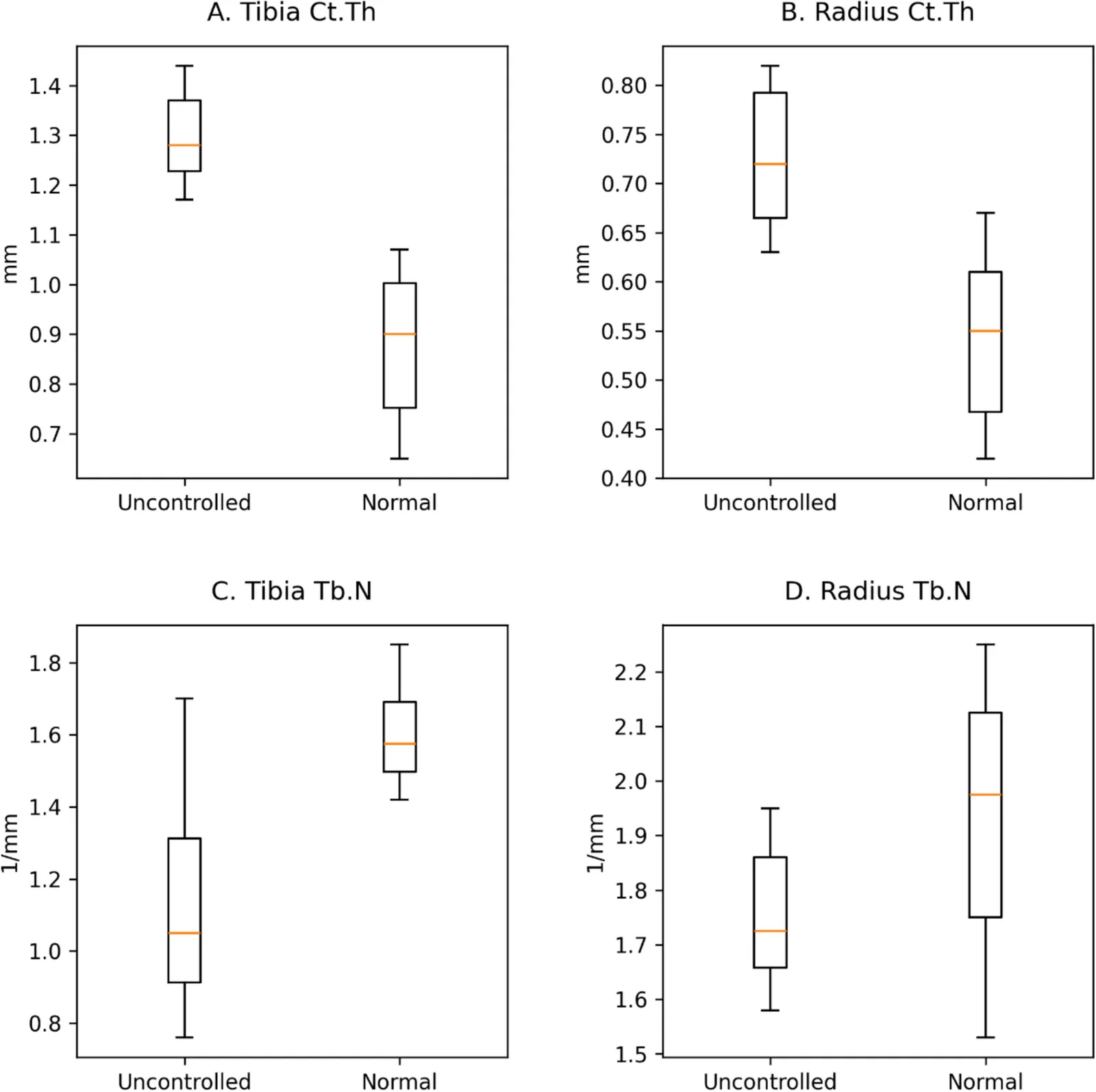
Cortical and trabecular bone microarchitecture according to serum phosphate status in adults with XLH. Participants with uncontrolled serum phosphate levels showed greater cortical thickness and lower trabecular number at both the tibia and radius compared with those with phosphate levels within the reference range. Box plots represent the median, interquartile range, and values within 1.5 times the interquartile range

At the radius, a similar pattern was observed, with slightly higher cortical vBMD and cortical thickness in patients with uncontrolled hypophosphatemia, whereas trabecular indices showed no consistent advantage. Trabecular number tended to be higher and trabecular separation lower in patients with normal serum phosphate, suggesting relatively more preserved trabecular structure in this subgroup (Fig. 1).

We compared patients with a longer disease duration (>40 years) versus a shorter duration (<40 years). Patients with longer disease duration exhibited impaired trabecular bone parameters, particularly at the tibia. After FDR correction, significant differences remained for tibial Tb.vBMD, Tb.N, Tb.Th, Tb.Sp and trabecular heterogeneity. Tibial Tb.BV/TV showed a borderline FDR-adjusted value. At the radius, similar but less pronounced numerical differences were observed, with moderate to large effect sizes, but they did not remain statistically significant after FDR correction (Table 7).

**Table 7:** HR-pQCT Parameters according to disease duration in XLH

Parameter	<40 years (*n *= 7)	>40 years (*n *= 9)	FDR *p*-value	Effect size (*δ*)
*Tibia*				
Total vBMD (mg HA/cm^3^)	253.0 (230.9–326.6)	208.9 (164.2–260.8)	0.231	− 0.39
Ct.vBMD (mg HA/cm^3^)	908.0 (891–938)	922.4 (833–973)	0.730	0.11
Tb.vBMD (mg HA/cm^3^)	119.3 (94–155)	45.3 (20–94)	**0.036**	− **0.78**
Tb.BV/TV	0.099 (0.079–0.133)	0.056 (0.017–0.079)	0.051	− **0.72**
Ct.Th (mm)	1.17 (0.95–1.89)	1.12 (0.65–1.26)	0.473	− 0.25
Tb.N (mm⁻^1^)	1.70 (1.06–1.85)	0.92 (0.57–1.42)	**0.036**	− **0.75**
Tb.Th (mm)	0.070 (0.059–0.074)	0.049 (0.041–0.063)	**0.036**	− **0.80**
Tb.Sp (mm)	0.514 (0.462–0.865)	1.201 (0.648–1.717)	**0.036**	**0.85**
SD.1/Tb.N (mm)	0.218 (0.212–0.485)	0.661 (0.407–0.943)	**0.036**	**0.83**
*Radius*				
Total vBMD (mg HA/cm^3^)	267.8 (262–347)	267.8 (208–288)	0.473	− 0.22
Ct.vBMD (mg HA/cm^3^)	890.8 (869–897)	900.0 (785–925)	0.614	0.18
Tb.vBMD (mg HA/cm^3^)	184.2 (130–207)	130.0 (76–154)	0.060	− **0.65**
Tb.BV/TV	0.154 (0.110–0.173)	0.108 (0.051–0.135)	0.060	− **0.68**
Ct.Th (mm)	0.76 (0.66–0.84)	0.62 (0.38–0.70)	0.051	− **0.71**
Tb.N (mm⁻^1^)	1.96 (1.65–2.27)	1.65 (1.18–1.81)	0.060	− **0.64**
Tb.Th (mm)	0.062 (0.056–0.076)	0.055 (0.043–0.075)	0.125	− 0.45
Tb.Sp (mm)	0.454 (0.365–0.531)	0.552 (0.478–0.807)	0.060	**0.62**
SD.1/Tb.N (mm)	0.181 (0.151–0.249)	0.249 (0.212–0.382)	0.060	**0.67**

Data are presented as median (interquartile range). Displayed *p*-values were adjusted using the Benjamini–Hochberg false discovery rate (FDR) procedure. Effect size is expressed as Cliff’s delta (δ). Significant values refer to FDR-adjusted *p*-values < 0.05

*vBMD* volumetric bone mineral density; *Tb.BV/TV* trabecular bone volume fraction; *Ct.Th* cortical thickness; *Tb.N* trabecular number; *Tb.Th* trabecular thickness; *Tb.Sp* trabecular separation; SD.1/*Tb.N* trabecular network inhomogeneity

Five patients presented with elevated bone alkaline phosphatase. We compared HR-pQCT parameters between patients with elevated versus normal bone alkaline phosphatase levels (Table 8). For the radius analysis, data were available for four patients with elevated BALP. Patients with elevated bone alkaline phosphatase showed a numerically distinct skeletal phenotype compared with those with normal levels.

**Table 8:** HR-pQCT parameters according to bone alkaline phosphatase status

Parameter	BALP elevated (*n *= 5)	BALP normal (*n *= 11)	FDR *p*-value	δ (Cliff’s)
*Tibia*				
Total vBMD (mg HA/cm^3^)	236.5 (218–301)	268.7 (197–345)	0.630	− 0.25
Ct.vBMD (mg HA/cm^3^)	894.7 (886–915)	915.5 (883–973)	0.558	− 0.30
Tb.vBMD (mg HA/cm^3^)	114.7 (78–139)	91.8 (44–149)	0.558	+ 0.35
Tb.BV/TV	0.096 (0.066–0.116)	0.077 (0.037–0.125)	0.605	+ 0.20
Ct.Th (mm)	1.10 (0.91–1.36)	1.17 (0.65–1.95)	0.660	− 0.10
Tb.N (mm⁻^1^)	1.37 (0.84–1.87)	1.42 (0.84–1.70)	0.688	− 0.05
Tb.Th (mm)	0.062 (0.053–0.085)	0.054 (0.044–0.073)	0.231	+ 0.50
Tb.Sp (mm)	0.749 (0.47–1.13)	0.648 (0.51–1.14)	0.405	+ 0.40
SD.1/Tb.N (mm)	0.382 (0.21–0.74)	0.407 (0.21–0.71)	0.700	− 0.05

Parameter	BALP elevated (*n *= 4)	BALP normal (*n *= 11)	FDR p-value	δ (Cliff’s)
*Radius*				
Total vBMD (mg HA/cm^3^)	258.9 (220–270)	279.6 (235–354)	0.216	− 0.65
Ct.vBMD (mg HA/cm^3^)	875.2 (775–908)	893.4 (791–931)	0.231	− 0.50
Tb.vBMD (mg HA/cm^3^)	133.7 (97–185)	154.4 (105–207)	0.216	− 0.60
Tb.BV/TV	0.111 (0.081–0.155)	0.129 (0.088–0.173)	0.216	− 0.55
Ct.Th (mm)	0.62 (0.45–0.66)	0.70 (0.54–0.88)	0.216	− 0.70
Tb.N (mm⁻^1^)	1.96 (1.41–2.37)	1.81 (1.44–2.33)	0.660	+ 0.15
Tb.Th (mm)	0.068 (0.056–0.068)	0.075 (0.055–0.081)	0.216	− 0.60
Tb.Sp (mm)	0.454 (0.35–0.67)	0.478 (0.37–0.65)	0.688	− 0.10
SD.1/Tb.N (mm)	0.181 (0.13–0.36)	0.212 (0.13–0.40)	0.660	− 0.15

Data are presented as median (interquartile range). *BALP* bone alkaline phosphatase. δ: Cliff’s delta. Comparisons between groups were performed using the Mann–Whitney U test, and displayed p-values were adjusted using the Benjamini–Hochberg FDR procedure. For radius analyses, HR-pQCT data were available for four patients with elevated BALP. Effect size was estimated using Cliff’s delta (δ), with values of 0.11, 0.28, and 0.43 representing small, medium, and large effects, respectively

At the radius, patients with elevated bone alkaline phosphatase showed numerically lower total vBMD, Tb.vBMD, Ct.Th and Tb.Th, with moderate to large effect sizes. However, these differences did not remain statistically significant after FDR correction.

In contrast, tibial findings were more heterogeneous. Although trabecular thickness appeared increased in patients with elevated alkaline phosphatase, this was accompanied by increased trabecular separation, suggesting a disorganized trabecular structure rather than true structural improvement.

These findings suggest a site-specific pattern, with non-weight-bearing bone potentially reflecting impaired bone quality, although the subgroup results should be interpreted cautiously after FDR correction.

## Discussion

Although DXA results suggested higher areal density in the spine and lower areal density in the 33% radius of the XLH group, bone assessment based on HR-pQCT proved more accurate, allowing us to define that adults with XLH treated with phosphate and calcitriol supplementation presented compromised bone volumetric density and microstructure in the trabecular and cortical compartments of both the distal radius and tibia, with emphasis on the impairment of the trabecular compartment.

The XLH group presented low levels of phosphorus (2.35 mg/dL ± 0.29), predominantly normal PTH (57.3 pg/mL ± 28.5), elevated FGF23 (252.7 KRU/L ± 325.3), and bone alkaline phosphatase at the upper limit of normal (24.5 mcg/L ± 15.7). In adults, only 50% of alkaline phosphatase is bone-specific, so although it has limitations for its applicability in diagnosis, it is useful as a sensitive marker of osteoblastic activity, helping to assess disease activity [14]. Thus, the increased ALP and BALP found in five XLH patients may reflect active disease. Conventional treatment usually fails to normalize serum phosphate in a sustained manner, and progressive increments in phosphate supplementation should not be a goal to avoid complications, such as secondary hyperparathyroidism. In our cohort, only three patients had elevated PTH at the study. However, some patients may have elevated PTH levels at diagnosis, probably related to PHEX deficiency, high FGF23 and calcitriol deficiency [15]. Conventional treatment partially improves biochemical and radiological changes but rarely normalizes all markers [14, 16].

DXA evaluation suggested controversial results in XLH patients, such as higher Z-score in the lumbar spine, no difference in the total hip, and lower Z-score in the radius, as compared to controls. Ectopic ossification and degenerative changes are common in adult XLH and may lead to overestimation of lumbar spine BMD. Although scans were reviewed for such artifacts, residual confounding cannot be completely excluded. The most consistent findings in the literature indicate that, in these patients, areal BMD measured by DXA tends to be increased in the lumbar spine, while the total femur shows normal or slightly reduced BMD, and the 33% radius has significantly reduced BMD, a region composed mainly of cortical bone [9]. These results could be attributed to the interference from anatomical factors, such as bone deformities, osteoarthritis and extra skeletal calcifications of the spine. The distinct mechanical load distribution in the axial region compared to the appendicular sites may influence the increase of the BMD at the lumbar spine [10, 17]. A study evaluating 66 patients with XLH (aged 29.6 ± 9.6 years) showed that the Z score of lumbar spine BMD was relatively high, although trabecular bone score (TBS) was normal [18]. Another study found higher TBS in XLH patients compared to non-XLH subjects, indicating a greater amount of trabecular bone in the lumbar spine, regardless of extraskeletal calcifications [19]. Although TBS in the lumbar spine tends to be normal-high and BMD is elevated in these patients, the increase in BMD does not indicate an improvement in bone quality, since DXA cannot reflect the microstructure of axial bone [18, 19].

Half of the group with XLH presented low BMD for age, all of them detected in the 33% radius: 60% of postmenopausal women, 50% of men, and 43% of premenopausal women. One postmenopausal woman with XLH was diagnosed with osteoporosis, also detected in the 33% radius. However, the diagnosis of osteoporosis should be made with caution and not based solely on areal BMD by DXA, due to the limitations of this test in this population, which may not reflect the real risk of fractures or the severity of osteomalacia. International consensus, including the expert group that published global guidelines for XLH, dictates that the diagnosis of osteoporosis should be based on clinical and radiological evidence of fractures, pseudofractures, and osteomalacia, and not solely on BMD values [14, 20].

The applicability of HR-pQCT in patients with XLH lies primarily in the detailed evaluation of volumetric bone density, not influenced by extraskeletal calcifications, and bone microarchitecture at distal radius and tibia. HR-pQCT allows the measurement of parameters such as cortical and trabecular volumetric bone mineral density, cortical thickness, cortical porosity, trabecular area, and bone stiffness. These findings are relevant because DXA can overestimate bone quality in XLH, while HR-pQCT reveals the true impairment of bone structure [14].

Evaluating density parameters by HR-pQCT in distal radius, we can observe a reduced total volumetric density, with a tendency towards reduced cortical density compared to the control group, with thinner cortical and trabecular thickness. Previous studies evaluating the radius using HR-pQCT show reduced total bone mineral density, reflecting the mineralization defect characteristic of XLH, primarily with reduced cortical density at this site. Cortical thickness is also reduced in both adults and children, and trabecular microarchitecture is impaired [18, 21–23].

In the distal tibia, XLH patients had lower total vBMD, Tb.vBMD, Tb.BV/TV and Tb.Th after FDR correction, while Tb.N, Tb.Sp and trabecular heterogeneity showed borderline FDR-adjusted values. These findings are consistent with impaired trabecular microarchitecture, including lower trabecular mass and thickness and greater trabecular separation and heterogeneity. It has already been demonstrated that XLH patients exhibit deterioration of the trabecular microarchitecture in the tibia, as reaffirmed by the present study [22]. These HR-pQCT findings reflect the mineralization defect characteristic of XLH, which is not fully corrected by conventional treatment and is also not well evaluated by DXA [22].

The main limitation of our study is the small number of XLH patients evaluated, which reflects the rarity of the disease and the difficulty of long-term follow-up. Nevertheless, the results suggest persistent bone involvement despite conventional treatment with phosphate salts and calcitriol, supporting the need for more sensitive skeletal assessment in adults with XLH.

We compared patients diagnosed and treated in the first 5 years of life with those with late diagnosis and treatment. There are no published studies that directly compare patients with XLH diagnosed and treated early versus those diagnosed and treated late in adulthood using HR-pQCT. The available literature presents separate studies in pediatric and adult populations, but without direct comparisons between groups with different treatment initiation times.

Our findings suggest that early diagnosis and treatment in XLH may be associated with partial preservation of trabecular bone microarchitecture, especially in the non-weight-bearing radius. Although these differences did not remain statistically significant after FDR correction, the large effect sizes observed for radius Tb.vBMD, Tb.BV/TV and Tb.Th support the biological relevance of this exploratory analysis despite the small sample size.

We compared the XLH group with hypophosphatemia versus controlled phosphorus, we did not observe statistically significant differences in HR-pQCT parameters. In the tibia of patients with uncontrolled hypophosphatemia, we found a tendency towards worse trabecular parameters (lower trabecular number and greater trabecular separation and heterogeneity), suggesting compromised trabecular microarchitecture, despite the relative preservation of cortical bone. At the radius a similar, though less pronounced, pattern was observed. Regardless of treatment status, patients with XLH exhibit characteristics consistent with these findings in HR-pQCT already demonstrated in previous studies [11, 18, 22]. Indeed, previous studies have demonstrated a significant improvement in cortical bone mineralization in the subgroup of treated patients [11]. However, it is important to emphasize that the average age of the XLH group should be considered, since the younger the patient, the shorter the exposure time to the deleterious effects on bone health. Although our group is undergoing conventional treatment, we recognize the difficulty of adherence to conventional treatment, even due to the tolerability of phosphate salts. Furthermore, patients who frequently use phosphate salts may stimulate the vicious cycle of the disease via PTH, worsening the characteristics of the cortical bone, increasing cortical porosity, and reducing cortical volumetric bone mineral density (vBMD) [22].

Patients with longer disease duration exhibited significantly impaired trabecular bone parameters compared to those with shorter disease duration, particularly at the tibia.

The literature suggests that trabecular involvement primarily affects the tibia more than the radius, and adults exhibit greater trabecular deficits in the radius than children, suggesting disease progression with age [11]. Although comparisons between children and adults suggest progression with disease duration, specific longitudinal studies comparing patients with different disease durations are limited in the current literature. Most studies are cross-sectional, comparing different age groups or pediatric versus adult patients.

Overall, these findings suggest a progressive decline in trabecular bone microarchitecture with disease duration in XLH, more pronounced in weight-bearing sites.

HR-pQCT analysis suggests that XLH primarily affects the trabecular compartment, with the tibia being more compromised than the radius. This may reflect differences in the ratio of trabecular to cortical bone between the two sites and in the differential response of these compartments to chronic hypophosphatemia.

The main explanation for the greater trabecular involvement in the tibia relates to the fact that it is a weight-bearing bone.

Studies in autosomal dominant hypophosphatemic rickets, which shares pathophysiological mechanisms with XLH, have demonstrated that FGF23 has a detrimental effect, especially on trabecular bone in weight-bearing sites [24].

XLH leads to hypophosphatemia and the accumulation of unmineralized soft tissue (osteoid tissue). This lack of mineralization affects the intrinsic stiffness of the bone and, under constant stress (such as when walking, for example), bone deformities arise. Furthermore, the osteocytes of patients with XLH exhibit periosteocytic lesions that form lacunae, causing an overestimation of the mechanical load. The tibia, being a weight-bearing bone, is subject to this atypical biomechanical stress, which can exacerbate trabecular defects. In fact, lower limb deformities lead to atypical mechanical loading, resulting in bone adaptations that may explain some of the differences observed between weight-bearing and non-weight-bearing sites [25].

Furthermore, it is worth noting that effective treatment of XLH appears to positively affect bone mineralization, mainly in cortical bone, which may contribute to the observed differences between sites, especially in studies that include treated and untreated patients.

Given the limited sample size, these findings should be interpreted cautiously, and adequately powered studies are warranted to clarify the relationship between phosphate control and bone microarchitecture in XLH.

As shown in Table 8. elevated bone alkaline phosphatase may reflect ongoing disease activity in XLH and was accompanied by numerical differences in bone quality parameters, particularly at non-weight-bearing sites, although these differences did not remain statistically significant after FDR correction. The apparent increase in trabecular thickness observed at the tibia may reflect accumulation of unmineralized osteoid rather than improved bone strength, consistent with osteomalacia. These results highlight the importance of assessing bone microarchitecture beyond density measures alone and suggest that bone alkaline phosphatase may be a useful surrogate marker of skeletal involvement in XLH. Previous studies have already demonstrated that optimal HR-pQCT settings correlate with lower levels of alkaline phosphatase, and have also shown that the radius presents specific impairment, and in some aspects more pronounced than the tibia, such as smaller cortical area, smaller cortical thickness, and greater cortical porosity [18, 26].

The relatively small sample size, inherent to the rarity of XLH, limits statistical power and increases the possibility of both type I and type II errors. To mitigate this limitation, effect sizes and FDR-adjusted analyses were performed and are presented in the tables. Therefore, the findings should be interpreted as exploratory and hypothesis-generating until confirmed in larger multicenter cohorts.

In conclusion XLH is a rare disease, with low clinical suspicion in many centers, which may delay diagnosis and effective treatment and negatively affect quality of life and autonomy. In our study, 33% radius areal BMD, but not spine or hip BMD evaluated by DXA, was more consistent with the bone deterioration detected by HR-pQCT. HR-pQCT identified lower total vBMD at both peripheral sites and impaired trabecular microarchitecture, particularly at the distal tibia. Exploratory subgroup analyses suggested that delayed diagnosis and longer disease duration may be associated with poorer trabecular microarchitecture, while elevated bone alkaline phosphatase showed a pattern of impaired bone quality, particularly at the radius; however, several subgroup findings did not remain statistically significant after FDR correction and should be interpreted cautiously. Considering that HR-pQCT is restricted to a limited number of research centers, assessment of the 33% radius by DXA may represent a useful complementary approach in adults with XLH. However, this suggestion should be interpreted cautiously given the exploratory nature of the study and the limited sample size.

## References

[1] Lambert A-S et al (2019) X-linked hypophosphatemia: management and treatment prospects. Jt Bone Spine 86:731–738 10.1016/j.jbspin.2019.01.012 30711691

[2] Mughal MZ (2011) Rickets. Curr Osteoporos Rep 9:291 10.1007/s11914-011-0081-0 21968816

[3] Al Juraibah F et al (2021) Diagnosis and management of X-linked hypophosphatemia in children and adolescent in the Gulf Cooperation Council countries. Arch Osteoporos 16:1–15 10.1007/s11657-021-00879-9 PMC7929956 33660084

[4] Laurent MR et al (2021) Consensus recommendations for the diagnosis and management of X-linked hypophosphatemia in Belgium. Front Endocrinol 12:641543 10.3389/fendo.2021.641543 PMC8018577 33815294

[5] Mao M et al (2020) Growth curves for children with X-linked hypophosphatemia. J Clin Endocrinol Metab 105:3243–3249 10.1210/clinem/dgaa495 PMC7448934 32721016

[6] Lecoq A-L et al (2020) Management of X-linked hypophosphatemia in adults. Metabolism 103:154049 10.1016/j.metabol.2019.154049 31863781

[7] Reid IR et al (1991) X-linked hypophosphatemia: skeletal mass in adults assessed by histomorphometry, computed tomography, and absorptiometry. Am J Med 90:63–69 10.1016/0002-9343(91)90507-t 1986592

[8] Beck-Nielsen SS et al (2010) Phenotype presentation of hypophosphatemic rickets in adults. Calcif Tissue Int 87:108–119 10.1007/s00223-010-9373-0 20524110

[9] Shore RM, Langman CB, Poznanski AK (2000) Lumbar and radial bone mineral density in children and adolescents with X-linked hypophosphatemia: evaluation with dual X-ray absorptiometry. Skeletal Radiol 29:90–93 10.1007/s002560050016 10741497

[10] Beck-Nielsen S et al (2013) High bone mineral apparent density in children with X-linked hypophosphatemia. Osteoporos Int 24:2215–2221 10.1007/s00198-013-2286-9 23389694

[11] Colares Neto G et al (2017) Evaluation of bone mineral density and microarchitectural parameters by DXA and HR-pQCT in 37 children and adults with X-linked hypophosphatemic rickets. Osteoporos Int 28:1685–1692 10.1007/s00198-017-3949-8 28194480

[12] Camacho PM et al (2020) American Association of Clinical Endocrinologists/American College of Endocrinology clinical practice guidelines for the diagnosis and treatment of postmenopausal osteoporosis—2020 update. Endocr Pract 26:1–46 10.4158/GL-2020-0524SUPPL 32427503

[13] Yong E-L, Logan S (2021) Menopausal osteoporosis: screening, prevention and treatment. Singap Med J 62:159 10.11622/smedj.2021036 PMC8801823 33948669

[14] Kamenický P et al (2024) X-linked hypophosphataemia. Lancet 404:887–901 10.1016/S0140-6736(24)01305-9 39181153

[15] Lecoq AL et al (2020) Hyperparathyroidism in patients with X-linked hypophosphatemia. J Bone Miner Res 35:1263–1273 10.1002/jbmr.3992 32101626

[16] Uday S et al (2021) Monitoring response to conventional treatment in children with XLH: value of ALP and rickets severity score (RSS) in a real world setting. Bone 151:116025 10.1016/j.bone.2021.116025 34052463

[17] Padlina I et al (2017) The lumbar spine age-related degenerative disease influences the BMD not the TBS: the Osteolaus cohort. Osteoporos Int 28:909–915 10.1007/s00198-016-3829-7 27900426

[18] Ni X et al (2022) Bone microstructure evaluated by TBS and HR-pQCT in Chinese adults with X-linked hypophosphatemia. Bone 160:116423 10.1016/j.bone.2022.116423 35439616

[19] de Paula Colares Neto G et al (2023) Evaluation of the trabecular bone score in 35 children and adults with X-linked hypophosphatemic rickets. J Bone Miner Metab 41:666–672 10.1007/s00774-023-01442-x 37418074

[20] Khan AA et al (2025) X-linked hypophosphatemia management in adults: an international working group clinical practice guideline. The J Clin Endocrinol Metab 110:170 10.1210/clinem/dgaf170 PMC12261105 40243526

[21] Wu Y et al (2024) Bone microarchitecture evaluated by HR-pQCT in Chinese adolescent and pediatric patients with X-linked hypophosphatemia. J Clin Endocrinol Metab. 10.1210/clinem/dgae782 39538411

[22] Shanbhogue VV et al (2015) Bone geometry, volumetric density, microarchitecture, and estimated bone strength assessed by HR-pQCT in adult patients with hypophosphatemic rickets. J Bone Miner Res 30:176–183 10.1002/jbmr.2310 25042721

[23] Cheung M et al (2013) Cortical and trabecular bone density in X-linked hypophosphatemic rickets. J Clin Endocrinol Metab 98:E954–E961 10.1210/jc.2012-4133 23533226

[24] Liu C et al (2023) Bone characteristics of autosomal dominant hypophosphatemic rickets patients. Bone 167:116602 10.1016/j.bone.2022.116602 36347435

[25] Nguyen-Khac V et al (2023) Changes in adipose bone marrow and bone morphology in X-linked hypophosphatemic rickets. Orthop Traumatol Surg Res 109:103529 10.1016/j.otsr.2022.103529 36565743

[26] Wu Y et al (2025) Bone microarchitecture evaluated by HR-pQCT in Chinese adolescent and pediatric patients with X-linked hypophosphatemia. J Clin Endocrinol Metab 110:2172–2181 10.1210/clinem/dgae782 39538411

